# Gall bladder wall thickening as non-invasive screening parameter for esophageal varices – a comparative endoscopic – sonographic study

**DOI:** 10.1186/s12876-018-0852-5

**Published:** 2018-08-02

**Authors:** Birgit Tsaknakis, Rawan Masri, Ahmad Amanzada, Golo Petzold, Volker Ellenrieder, Albrecht Neesse, Steffen Kunsch

**Affiliations:** Department Gastroenterology and Gastrointestinal Oncology, University Medical Centre Goettingen Georg-August-University, Robert-Koch-Str. 40, 37075 Goettingen, Germany

**Keywords:** Esophageal varices, Gall bladder wall, Cirrhosis, Liver disease, Portal hypertension, Non-invasive parameter, Ultrasound parameter, Child-Pugh-score

## Abstract

**Background:**

The mortality due to hemorrhage of esophageal varices (EV) is still high. The predominant cause for EV is liver cirrhosis, which has a high prevalence in Western Europe. Therefore, non-invasive screening markers for the presence of EV are of interest. Here, we aim to investigate whether non-inflammatory gall bladder wall thickening (GBWT) may serve as predictor for the presence of EV in comparison and combination with other non-invasive clinical and laboratory parameters.

**Methods:**

One hundred ninety four patients were retrospectively enrolled in the study. Abdominal ultrasound, upper endoscopy and blood tests were evaluated. GBWT, spleen size and the presence of ascites were evaluated by ultrasound. Platelet count and Child-Pugh-score were also recorded. The study population was categorized in two groups: 122 patients without esophageal varices (non EV) compared to 72 patients with EV were analyzed by uni-and multivariate analysis.

**Results:**

In the EV group 46% showed a non-inflammatory GBWT of ≥4 mm, compared to 12% in the non-EV group (*p* < 0.01). GBWT was significantly higher in EV patients compared to the non-EV group (mean: 4.4 mm vs. 2.8 mm, *p* < 0.0001), and multivariate analysis confirmed GBWT as independent predictor for EV (*p* < 0.04). The platelets/GBWT ratio (cut-off > 46.2) had a sensitivity and specificity of 78 and 86%, PPV 76% and NPV of 87%, and ROC analysis calculated the AUC of 0.864 (CI 0.809–0.919)**.**

**Conclusions:**

GBWT occurs significantly more often in patients with EV. However, because of the low sensitivity, combination with other non-invasive parameters such as platelet count is recommended.

## Background

The prevalence of liver cirrhosis is estimated to be between 0.15 and 0.3% in European countries [1]. The main causes are alcohol abuse, infection with viral hepatitis B and C as well as autoimmune liver diseases [2]. A clinically relevant complication is the development of portal hypertension with all its clinical consequences such as ascites, spontaneous bacterial peritonitis and development of portosystemic collaterals. A progression rate of 12% has been reported for esophageal varices (EV) [3]. Although the mortality of variceal hemorrhage has declined in the last decades, it is still very high with a six-week-mortality of up to 37% [4], and a high recurrence rate after the first bleeding incident [5]. Although repeated endoscopic controls of patients with an advanced liver fibrosis or liver cirrhosis are justified, it is an invasive diagnostic procedure with its own risks, and it is not always widely available in countries with lower health care standards. Therefore, non-invasive predictors for portosystemic collaterals are of high interest. Notably, the venous blood is drained from the gall bladder in part via small vessels directly into the liver. An additional venous blood drain flows via small veins towards the cystic duct and then with vessels from the common bile duct terminating in the portal venous system [6].Therefore, the gall bladder should be directly affected by portal hypertension causing a thickened gall bladder wall due to impaired venous drainage. Here, we aim to determine whether non-inflammatory gall bladder wall thickness (GBWT) correlates with the presence of EV. To this end, we performed a retrospective endoscopic-ultrasonographic study correlating the presence of EV and GBWT with other non-invasive parameters for liver disease and portal hypertension.

## Methods

In this study we retrospectively included all patients with chronic hepatic disease, who received an ultrasound of the abdomen either as an inpatient or outpatient in the Department of Gastroenterology and Gastrointestinal Oncology of the University Hospital of Goettingen between April 2015 and January 2016. Patients who had a cholecystectomy or complained of upper abdominal pain were excluded from the study. Gall stones and single gall bladder polyps without symptoms were no exclusion criteria. Of all patients who also had a documented upper endoscopy (median time interval 147 days), the following parameters were evaluated by ultrasound: The thickness of the gall bladder was measured twice after overnight fasting at two different locations and an average value was calculated. The spleen length was measured from a left lateral cross section. The diameter of the portal vein, the portal blood flow velocity and the liver size were measured. Ultrasound and endoscopy examinations were performed by experienced Gastroenterology trainees (> 3 years experience) and senior Gastroenterology consultants. The presence or absence of ascites was recorded. Additionally, clinical parameters such as the Child-Pugh-classification, laboratory results and upper endoscopic findings (presence of EV graded according the classification of Paquet) were obtained. Using the results of the cranio-caudal spleen diameter, gall bladder wall diameter and laboratory results, we calculated the ratio of platelet count to spleen diameter and the ratio of platelet count to gall bladder wall thickness. The statistical analysis was performed using the Mann Whitney U and Chi square test. Furthermore, variables with a *P* value < 0.1 from univariate analysis entered the multivariate binary logistic regression analysis and (receiver operating characteristic) ROC analysis was performed by SPSS Version 25 Mac OS. Since patient data were collected retrospectively and did not influence the diagnostic or therapeutic management of the patients, the ethic committee at the University Medical Centre Goettingen, Germany, was informed in written form about the study prior to data collection but did not request a separate ethical votum (24/7/15AN).

## Results

### Patient characteristics

**Fig. 1:**
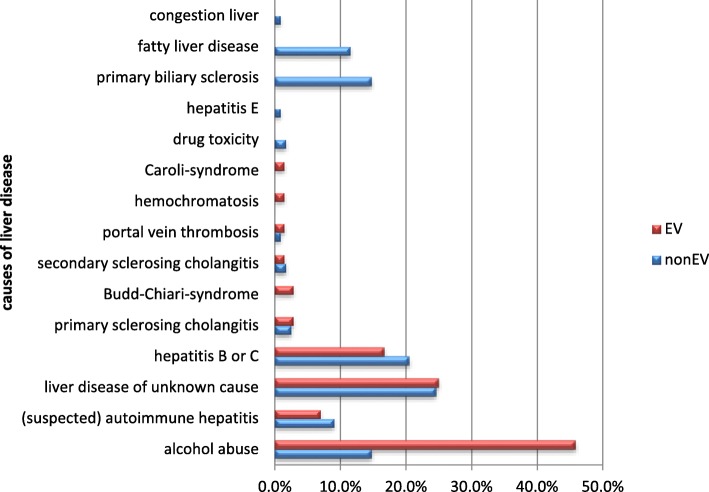
Underlying liver diseases of study cohort (*n* = 194 patients)

A total of 194 patients were included in this study, of whom 84 were female and 110 were male. The average age of the patients at time of ultrasound examination was 57 years (range: 17–85 years). The main cause of hepatic disease was alcohol abuse (*n* = 51), followed by unknown cause of liver illness (*n* = 38) and viral hepatitis B and C (*n* = 35). The underlying liver diseases are summarized in Fig. 1. The patients were divided into two groups: 122 patients without EV (referred to as “non-EV” group) and 72 patients with EV in endoscopic diagnostic examination (referred to as “EV” group). Of those with EV 31 patients had 1° varices, 32 patients had 2° and further 9 patients had 3° varices. Interestingly, male patients were significantly more often represented in the EV group (73.6% EV group vs. 46.7% non-EV group; *p* < 0.001), potentially reflecting the high percentage (46%) of patients with alcohol abuse in the EV group. Histology of the liver was available in 53% of all patients (*n* = 102), and in those, cirrhosis was confirmed in 63% (*n* = 22) in the EV-group, and 19% (*n* = 13) in the non-EV group. As expected, hypertensive gastropathy, advanced Child Pugh Score and presence of ascites occurred significantly more frequently in the EV group. The patient characteristics disease severities are summarized in Table 1.

**Fig. 2:**
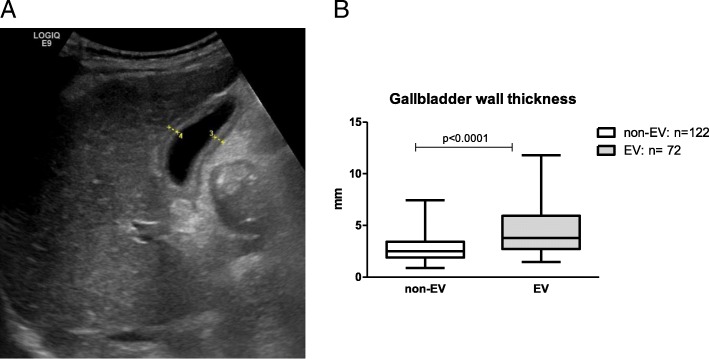
**a** Sonographic measurement of gall bladder wall thickness (GBWT) at two different locations. **b** GBWT is significantly different in patients with esophageal varices (EV) compared to non-EV patients (*p* < 0.0001, Mann Whitney-U test)

**Table 1:** Patient characteristics (comparison of group non-EV without esophageal varices and group EV with esophageal varices found by upper endoscopy)

Parameter	non-EV *n* = 120	EV *n* = 72	*p*-value
Male sex	47%	74%	< 0.001
Age (mean)	57 ± 14	57 ± 13	ns
Hypertensive gastropathy	14%	74%	< 0.0001
Child-Pugh-Classification A	92%	50%	< 0.0001
Child-Pugh-Classification B	3%	26%	< 0.0001
Child-Pugh-Classification C	5%	23%	< 0.001
presence of ascites	6%	44%	< 0.0001

### Ultrasonographic findings

In the EV group 46% of patients showed a non-inflammatory (absence of clinical and laboratory signs of acute cholecystitis) GBWT of ≥4 mm, compared to 12% in non-EV group (*p* < 0.01). GBWT was significantly higher in EV patients compared to the non-EV group (mean: 4.4 mm vs. 2.8 mm, *p* < 0.0001) Fig. 2a, b. The median of non-EV was lower with 2.5 mm than the median of 3.8 mm in the EV group. A more detailed analysis of the EV group revealed that there was no significant difference between first, second and third degree EV subgroups with an average thickness of 4.3 mm, 4.5 mm and 4.2 mm, respectively.

The spleen size as additional ultrasound parameter is also indicative for portal hypertension. In our cohort, the average spleen length was significantly higher in the EV group compared to the non-EV-group (138 mm vs. 113 mm; p < 0,001; Table 2). The portal vein diameter was also significantly higher in the EV group (12.4 mm vs. 11.6 mm; *p* = 0.045; Table 2). Further parameters measured by ultrasound such as average portal vein blood flow velocity, gall bladder length, and gall bladder diameter did not show any significant difference between the two groups (Table 2).

**Table 2:** Sonographic, clinical and laboratory findings in patients without esophageal varices (non-EV = 122) and endoscopically confirmed varices (EV = 72)

Parameter	non-EV *n* = 120	EV *n* = 72	*p*-value
Gall bladder wall thickness	2.8 ± 1.2 mm	4.4 ± 2.1 mm	**< 0.0001**
Gall bladder length	61.6 ± 17.6 mm	61.1 ± 21.8 mm	0.867
Gall bladder diameter	25.0 ± 8.6 mm	27.1 ± 10.0 mm	0.128
Liver size in MCL	13.8 ± 2.1 cm	14.7 ± 2.6 cm	**0.015**
Spleen diameter	112.9 ± 23.9 mm	138.0 ± 28.2 mm	**< 0.0001**
Portal vein diameter	11.6 ± 2.1 mm	12.4 ± 2.9 mm	**0.045**
Portal vein velocity	18.0 ± 3.9 cm/s	18.0 ± 5.8 cm/s	0.968
INR	1.09 ± 0.38	1.39 ± 0.46	**< 0.0001**
Platelet count	226.6 ± 85.9 × 1.000/μl	128.1 ± 99.2 × 1.000/μl	**< 0.0001**

*INR* International ratio; *MCL* Medioclavicular line

Significant are all values <0.05

### Biochemical analysis

The platelet count was significantly lower in the EV group (128.000/μl EV group vs. 227.000/μl non-EV group; *p*-value < 0.001; Table 2).

The average value of the INR differed significantly between the two groups with 1.09 ± 0.38 in the non-EV group and 1.39 ± 0.46 in the EV group (p-value < 0.0001). Sensitivity, specificity, positive and negative predictive value were calculated for single parameters regarding the presence of EV and showed sensitivities ranging between 40 and 70% (Table 3). In particular, the sensitivity of GBWT of ≥4 mm for the presence of EV was 46%, while the specificity was 89%. The positive predictive value was 70% and the negative predictive value 73%.

**Table 3:** Sensitivity, specificity, positive and negative predictive value for the presence of esophageal varices using gall bladder wall thickness (GBWT) ≥4 mm, spleen length (≥130 mm), ascites, thrombocytes (< 160.000/μl) and Child-Pugh classification

Parameter	GBWT (≥4 mm)	Spleen (≥130 mm)	Thrombocytes (< 160.000/μl)	Ascites	Child-Pugh B or C
Sensitivity	46%	62%	69%	44%	50%
Specificity	89%	81%	78%	94%	92%
Positive predictive value	70%	67%	64%	82%	78%
Negative predictive value	73%	78%	81%	74%	76%

Using multivariate analysis by logistical regression including sex, Child-Pugh score, GBWT, liver size, spleen diameter, International Normalized Ratio (INR), platelet count, ascites and portal vein diameter, we show that GBWT, ascites, platelet count and spleen diameter are independent predictors of EV (Table 4).

**Table 4:** Results of logistic regression analysis for prediction esophageal varices

Logistic regression for prediction esophageal varices						
	B	S.E.	df	*p*	OR = Exp(B)	95% CI for OR
GBWT	−0.323	0.155	1	0.037	0.724	0.534–0.977
Spleen diameter	−0.023	0.009	1	0.007	0.977	0.961–0.994
Platelet count	0.009	0.003	1	0.001	1.009	1.004–1.015
Ascites	1.674	0.717	1	0.019	5.336	1.310–21.731

*B* Regression coefficient; *S.E*. Standard error; *df* Degree of freedom; *OR* Odds ratio; *GBWT* Gall bladder wall thickness

**Fig. 3:**
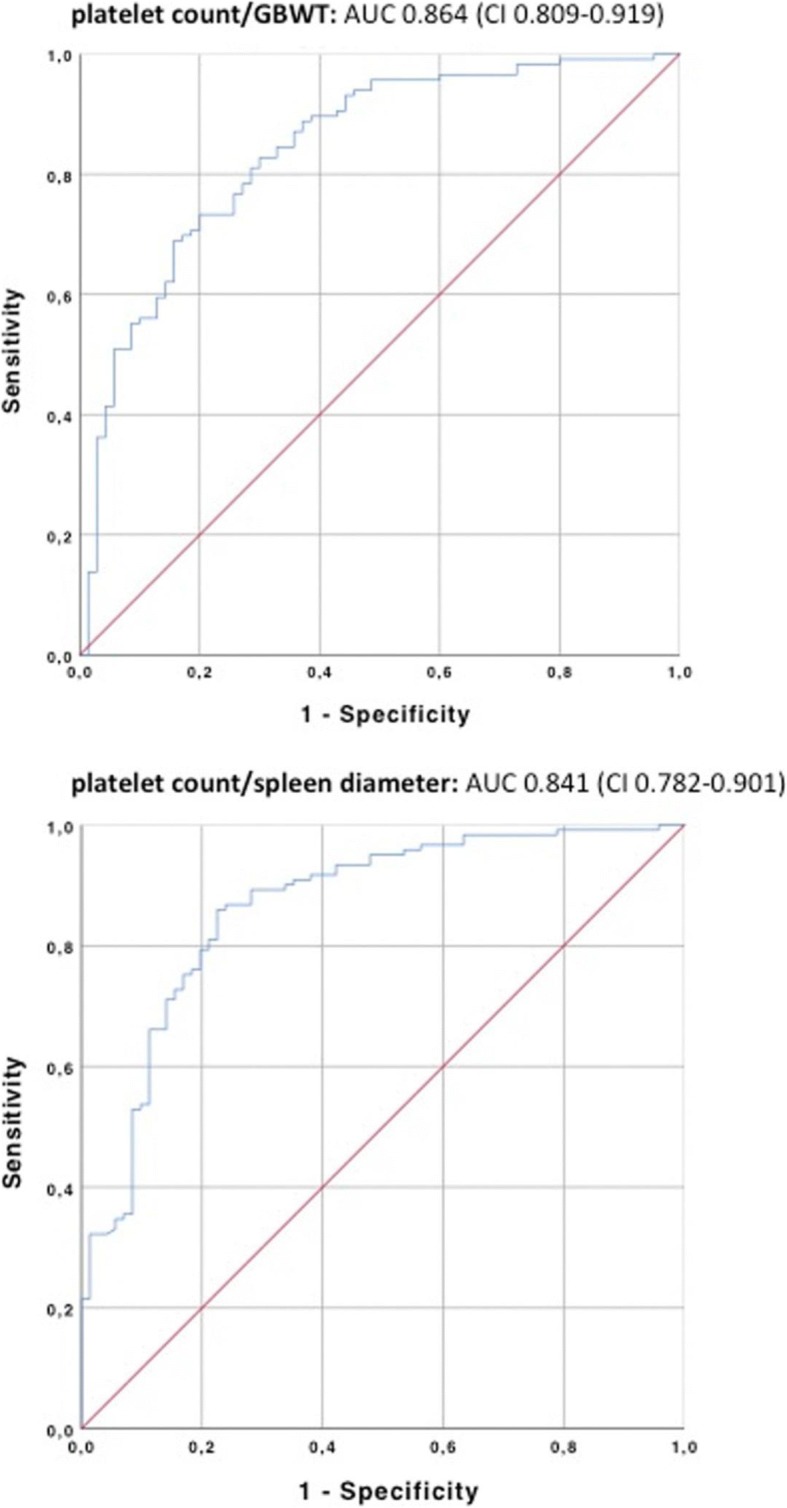
Receiver operating characteristic (ROC) analysis and area under the curve (AUC) Top platelet count/GBWT ratio. Bottom platelet count/spleen diameter ratio

However, platelet count/GBWT ratio (cut-off > 46.2) achieves a sensitivity of 78% and a specificity of 86%. The positive predictive value is 76% and the negative predictive value 87% (Table 5). Using our dataset, ROC analysis showed that the platelet count/GBWT ratio performed at a comparable level (area under the curve (AUC) 0.864 (confidence interval (CI) 0.809–0.919)) to the platelet count/spleen diameter ratio of 909 (AUC 0.841 (CI 0.782–0.901)) that was reported by Giannini et al. [7] (Fig. 3).

**Table 5:** Sensitivity, specificity, positive and negative predictive value and area under the curve (AUC) for the presence of EV using the combination of platelet count/GBWT ratio (> 46.2), and platelet count/spleen diameter 909 as described by Giannini et al. [7]

Parameters	Platelet count/GBWT (> 46.2)	Th/Spleen (< 909)
Sensitivity	78%	63%
Specificity	86%	87%
Positive predictive value	76%	75%
Negative predictive value	87%	80%
Area under the curve	0.864 (CI 0.809–0.919)	0.841 (CI 0.782–0.901

## Discussion

Patients with compensated liver cirrhosis have a chance of up to 40% to develop EV [8]. To avoid hemorrhage from EV, it is recommended to perform an upper endoscopy as soon as there are signs for the presence of liver cirrhosis in patients [9, 10]. Therefore, many patients undergo upper endoscopy although they do not require treatment of EV (e.g. ligation) according to endoscopic classifications. While diagnostic gastroscopy itself is of low risk, low platelet counts as well as impaired coagulation parameters increase the risk of complications. Most patients prefer sedation during the procedure which is associated with additional risks. Therefore, more accurate non-invasive parameters for the presence of EV could be a valuable and clinically relevant tool. We based our study on non-invasive, standard diagnostic tests, which are routinely performed in patients with chronic liver disease: ultrasound, clinical and laboratory results were evaluated in terms of prediction of EV.

Because of its portal-venous blood supply, we assumed that the GBWT may predict the presence of portal hypertension and EV. An interesting study by Maruyama et al. also reported a lower sensitivity of 62% regarding the detection of large esophageal varices using the platelet count to spleen diameter ratio in 229 cirrhosis patients. In this study, the authors showed that a diameter of the left gastric vein -as a non-variceal collateral- of more than 5.35 mm had a sensitivity of 90% and a specificity of 62% for presence of large esophageal varices. Its sonographic detection was associated with a sensitivity of 84% for any esophageal varices and a sensitivity of 100% for large varices [11]. However, further prospective studies are required to assess the value of portal vein velocity as non-invasive parameter for the presence of esophageal varices.

A small Chinese study showed a correlation between portal vein velocity and GBWT supporting the hypothesis that GBWT could also predict the presence of EV [12]. From a pathophysiological point of view, GBWT may be a microcirculatory driven event caused by impaired portalvenous outflow before significant changes in portal vein velocity occur. However, the development of GBWT may also be caused by other factors such as the serum-ascites albumin gradient (SAAG) [13].

Several studies have investigated non-invasive parameters as predictors for the presence of EV. A platelet count to spleen diameter ratio of 909 and less was associated with EV [7]. The enlarged spleen is caused by portal hypertension and low platelets were also associated with a lowered thrombopoetin serum level due to reduced liver function [14]. Chen performed a meta-analysis to confirm the usefulness of this ratio and calculated a summative sensitivity of the ratio of 84% with a specificity of 78% to predict EV. The sensitivity of this ratio was also influenced by etiology of the liver disease with a sensitivity of 92% in viral liver cirrhosis [15]. Using the platelet count to spleen diameter ratio as previously described [7], the sensitivity was somewhat lower with our dataset. The reasons might be the greater variety of causes for liver disease in our cohort than in previous evaluations. Another non-invasive method is the use of computed tomography (CT) imaging with a sensitivity of 90% and a specificity of 72% for the detection of EV [16]. The higher sensitivity is traded against higher costs, exposure to irradiation, and the use of contrast agents. Other non-invasive measurements such as liver stiffness measurements are promising but further studies need to be performed. Meta-analysis of data so far collected by using transient elastography (FibroScan®) showed lower prognostic values for liver stiffness [17]. A meta-analysis of studies using different modes of elastography techniques to measure spleen stiffness showed heterogeneous results to detect EV [18]. The sensitivity of liver stiffness was 84% in predicting any varices, compared to 78% using the stiffness of the spleen as parameter. The specificity of the spleen stiffness was higher when compared to liver stiffness (76% versus 62%) [18] [16]. The use of capsule endoscopy to detect EV is also discussed in the literature [19] but high costs and its semi-invasive nature need to be kept in mind. Because of those limitations of the aforementioned non-invasive methods, the use of GBWT could represent a novel and feasible clinical marker for the detection of EV.

Alcantara previously published a cut-off value of 4.35 mm for a thickened gall bladder wall and found a sensitivity of 60% and a specificity of 90% regarding the presence of EV in pediatric patients [20]. The sensitivity was higher than in our study, although a higher value was used as cut-off. Other reasons for this difference might be the different patient cohorts, since de Alcantara based his study on data from children with various causes of cirrhosis such as biliary atresia and autoimmune hepatitis [20]. In our study we used data from adult patients with chronic liver disease and common causes for cirrhosis in Western Europe, but lack of histological confirmation in almost half of them. The cut-off-value of 4 mm was arbitrarily set for univariate analysis and seems reasonable since a lower value could be measured in individuals that did not fast overnight with a higher rate of falsely positive cases. A higher cut-off value would lower sensitivity.

In addition, a lowered velocity within the portal vein seems reasonable in the presence of portal hypertension and esophageal varices, but existing data are still conflicting. An Indian study of sonographic parameters predicting esophageal varices in 56 patients showed a significant difference of mean portal vein velocity. In presence of esophageal varices the mean velocity was 14.77 cm/s and in absence of varices 17.66 cm/s [21]. However, Li et al. did not report a significant difference in portal vein velocity between patients with and without esophageal varices. The mean velocity was 15.3 cm/s in healthy individuals, 14.2 cm/s in patients with first degree varices, 13.1 cm/s in second degree varices and 12.0 cm/s in third degree varices [22]. In our study there was no difference in average portal vein velocity with 18.0 cm/s in both groups.

Our study has several limitations. First, ultrasonography and endoscopy were not always performed within a few days and may have biased results in case of rapidly changing endoscopy or ultrasonography findings. Furthermore, retrospective data collection could not establish a clear cause for chronic liver disease in almost 20% of patients. Secondly, although performed by experienced Gastroenterology trainees and consultants, GBWT measurements were performed by only one examiner, and inter-observer variability could thus not be accounted for. Third, we could not detect significant differences between small and large EV most likely due to the relatively low number of 3° EVs (*n* = 9) in the EV cohort.

## Conclusions

To conclude, GBWT may improve the non-invasive monitoring of liver disease patients to assess the risk for the presence of EV. However, a distinction between different severity grades of EV was not possible with the cut-off values used in our study. To improve upon the predictive value of GBWT, the combination with additional non-invasive parameters such as the platelet count is recommended.

## Abbreviations

AUCa: Area under the curve
CI: Confidence interval
EV: Esophageal varices
GBWT: Gall bladder wall thickening
INR: International normalized ratio
ROC: Receiver operating characteristic
